# Suggestions on leading an academic research laboratory group

**DOI:** 10.1515/biol-2022-0061

**Published:** 2022-06-15

**Authors:** Frank C. Church

**Affiliations:** Department of Pathology and Laboratory Medicine, The University of North Carolina School of Medicine, Chapel Hill, NC 27599, USA

**Keywords:** laboratory research group, academic laboratory, principal investigator, memories of laboratory group, hemostasis and thrombosis, graduate school, legacy

## Abstract

This commentary is about running an academic research laboratory group, including some reflections, memories, and tips on effectively managing such a group of scientists focused on one’s research. The author’s academic career has spanned from 1982 to 2022, including postdoctoral research associate through the rank of professor with tenure. Currently, the author is in the final year of 3 years of phased retirement. One must be willing to work hard at running a research laboratory. Also, stay focused on funding the laboratory tasks and publishing one’s work. Recruit the best people possible with advice from the collective laboratory group. Laboratory group members felt more like they were a part of a collective family than simply employees; however, what works best for the researcher is what matters. Several other points to discuss will include managing university roles, recruiting laboratory personnel, getting recognition, dealing with intellectual property rights, and publishing work. In closing, there are many more positives than negatives to leading a research laboratory group. Finally, one cannot replace the unforgettable memories and the legacy of a research laboratory group.

## 1 Introduction

*“When the right ideas emerge, a completely indescribable process of high intensity comes to pass in the soul of the person who sees them.” Werner Heisenberg, The Meaning of Beauty in Exact Natural Science, 1971.*

Graduate school does not prepare one for the leadership role that can occur in one’s career. One’s postdoctoral research fellowship gets one closer to the real deal, but not really. What type of position is the author describing? The job is to be the director/principal investigator (PI) of a research laboratory in a university. The role of the PI exists to provide the overall direction of the laboratory, to oversee that progress is made; and the harmony of the laboratory group is entirely under the control of the PI. A difference between university and industry types of research settings is that in academics, one is expected to support the salary/stipend of the people in one’s laboratory, primarily graduate research assistants, postdoctoral research fellows, and research technicians. Another difference is the ability to collaborate and help train junior researchers and undergraduate students that attend your institution and other universities. One does this by obtaining research funds for the laboratory to exist. Essentially, managing a university laboratory is like a small business, one operates on a budget and one does the best under these financial circumstances.

The life course of a PI depends somewhat on leadership style, research focus, personal beliefs/characteristics, and one’s life history. The author’s diagnosis of Parkinson’s disease made living well and staying healthy a higher priority than being a PI, thus, leading to the phased retirement plan that included giving up the research laboratory. However, usually, the laboratory’s longevity ultimately is provided by productivity from the laboratory group (publications and funding), which does include successfully training and graduating students in one’s laboratory. The author kept a laboratory group running for 30+ years amidst numerous US federal budget crises. Furthermore, the author typically had a laboratory group that consisted of (on average) two or three graduate students, one or two postdoctoral fellows, several undergraduates, and one technician. Finally, this commentary is one scientist’s view of his academic career, including several pointers for those just getting started forming a laboratory group. However, it also consists of a lot of great memories.

## 2 Academic research

### 2.1 Research philosophy

*“Imagination is more important than knowledge.” Albert Einstein*

To begin as a PI, one needs a statement (or philosophy) of research describing the overall goals of the laboratory. The author’s research philosophy was to provide a supportive environment for students (undergraduate, medical, and graduate) to perform biomedical research, to allow postdoctoral fellows the opportunity to direct the entire scientific process, and to strive for a caring laboratory family. The author believed that research should be considered an “apprenticeship,” and providing this training was paramount to his career goals. His basic science research was in protein structure–activity relationships, in the molecular basis of disease, and in trying to bridge these two endeavors to develop clinical therapeutics. His overall goal was to be an effective mentor and provide scholarship and education to everyone in this laboratory.

Your background and training provided one with the opportunity to explore directly the science of one’s future. Be both specific and broad in these depictions. The author’s *academic research* was historically centered on serine proteases and serine protease inhibitors (Serpins). The pathological processes he studied were venous thrombosis (hemostasis, thrombosis, and fibrinolysis) and cancer (breast cancer cell migration/chemotaxis/signaling). Today, his scholarship focuses on educational strategies [1,2,3,4], and he writes reviews on various aspects of Parkinson’s disease [5,6,7,8,9,10]. Historically, the author’s laboratory group funding was by the following federal agencies and national organizations: National Institutes of Health (Heart, Lung, and Blood Institute; National Institute of Aging; and National Institute of Neurological Disorders and Stroke); American Heart Association; and Susan G. Komen for the Cure.

### 2.2 Academics requires one to wear multiple hats

*“Research is to see what everybody else has seen, and to think what nobody else has thought.” Albert Szent-Gyorgyi*

Balancing life in academics can be a struggle due to the many hats (i.e., responsibilities) one typically is asked to wear in such a role. Scientist, teacher, mentor, and administrator are typical responsibilities. Many can get by with primarily running their laboratory group. Some enjoy teaching and add it to their portfolio. Others deem administrative work to be something they enjoy. All along, as you progress, many will ask for advice, so mentoring is also a feature of your job duties. Frequently, one ends up doing all these tasks. Balancing their responsibilities can be challenging at times. Managing it all can be stressful; however, one reaches a point in their career where what works best for the PI will become evident, and these roles will be fully and well defined.

Here are comments on what it takes to be an effective researcher, teacher, administrator, and mentor:


**Effective researcher** – implies that the laboratory director is willing to take on the challenges of leading a laboratory group, one will never give up trying to keep the laboratory funded and well-focused, one will see out-front several years to where the group currently is located because the next grant proposal depends on this foresight and planning.
**Effective educator** – important that the faculty member cares about the students, their welfare, mental health, and education; commitment to preparation and organization; knowledge and attention to the material; clarity of the message; and passion and enthusiasm for the entire process.
**Effective administrator** – essential to manage time effectively and understand the role and outcome expected; likely one will have assigned administrative assistance that will play a role in one’s (hopeful) successful outcome, and the work must get done.
**Effective Mentoring** – requires that the faculty member truly cares, obligates time to carefully listen, presumes one respect each person, infers that one is responding to and enabling their dream, and stipulates that one’s office door is always open and phone/email always on.

### 2.3 Recruiting personnel to the laboratory

*“It is a good morning exercise for a research scientist to discard a pet hypothesis every day before breakfast. It keeps him young.” Konrad Lorenz*

Although it is your laboratory, the laboratory group will work with the new technician and postdoctoral research associate daily, up close. The PI will likely deal with them one-on-one or in a larger group setting. Therefore, when interviewing, let the laboratory group have equal time without the director present to interview and meet any new person. Disasters may occur if one just hires someone, and the laboratory group cannot get along with that person at all. It matters a lot. The laboratory group will also get a chance to work with the rotating students, which is good. Moreover, having a talented and well-intentioned laboratory group will provide an excellent working environment for the rotating student. Gather information as the student goes on through the rotation; the laboratory group insight will be instrumental in helping one decide to recruit this student to one’s laboratory for their PhD (remember, earning a PhD usually takes 4–5 years). Do not forget you are the *brain* of the laboratory, but your laboratory group is your *heart*; they both need to be present to work well together, especially when recruiting a new person to the laboratory group.

### 2.4 Laboratory group personality

*“Try first to be a man of value; success will follow.” Albert Einstein*

The laboratory group consists of a variety of personalities, it is expected. They will sometimes be best friends, sometimes not at all. However, the author looked for recruitments to the laboratory, first, not to be disgruntled by science, and second, those willing to be team players. The best candidate likely would be described as having a hard-working attitude. They were both intelligent and independent thinkers, with ‘good hands’ at the laboratory bench, managing time well, and writing with ease. It takes unique individuals to bring out the best qualities in one’s laboratory. Uniformity may work for some PIs, but various life experiences from the candidates bring out the best in the laboratory group and its collective personality. The diversity provided by each person recruited to the laboratory could yield a new perspective or paradigm, which could ultimately provide a new path of discovery in the laboratory. Each person in the laboratory group was intelligent, but when one thinks about it, the application and use of their knowledge truly helped each person achieve success. So, yes, recruit intelligent people, but recruit those best able to take advantage of their intelligence. Furthermore, recruit people who can tolerate the frustration frequently found in science.

### 2.5 Publications throughout one’s career

*“Science, my lad, is made up of mistakes, but they are mistakes which it is useful to make, because they lead little by little to the truth.” Jules Verne*

The primary issue is how much time and effort one put into gathering the data and writing the article; in other words, what was the total time expended? If one’s article was published in an appropriate frontline journal, it was considered a success. Alternatively, some work was never meant for a frontline journal, yet it still deserved to be published and would represent the laboratory group well and those writing the article. Furthermore, an occasional article did not make it into what we considered the best journal for the target audience. This is where the most significant lessons were learned from the scientific process to the publication; while we might consider it somewhat of a letdown, it still eventually got published. Finally, on rare occasions even when the data were very believable, the interpretation and understanding of the questions being asked were just not convincing enough at that time, and the work was not published.

Shown in Table 1 are the numbers of publications at every phase of the author’s career, beginning with undergraduate and finishing up in the phased-retirement time. The results suggest a (relatively) steady flow of publications, notwithstanding the quality of the work, progressing through the academic ranks. The author’s initial goal on publications was to aim high for the first choice of articles in his field of research (e.g., The Journal of Biological Chemistry; Biochemistry; Proc. Natl. Acad. Sci. USA; Blood; Arteriosclerosis, Thrombosis, and Vascular Biology; J. Thromb. Haemost.; Biochimica Biophysica Acta; or Experimental Cell Research).

**Table 1:** The author’s publications aligned with academic rank

degree/work stage	Years	Number of publications
BS	1971–1975	0
MS	1976–1978	2
PhD	1978–1982	6
Postdoctoral res. assoc.	1982–1985	10
Res. assistant prof	1985–1986	3
Assistant professor	1987–1993	34
Associate professor	1994–1998	28
Professor	1999–2019	54
Phased-retirement	2019–2022	10
Articles in preparation	2022–present	6
Total = 153		

There are many ways to evaluate success in science, such as publishing in journals with high impact factors. Another way is calculating what is called the *h-index* from Google Scholar. The *h-index* for the author for all years is 49. The *h-index* is defined as the maximum value of *h* such that the given author/journal has published at least artilces that have each been cited at least *h* times [11]. Therefore, what is an excellent *h*-index value? The creator of the *h*-index, Jorge Hirsch, summarizes that an *h*-index of 20 is good, an *h*-index of 40 is outstanding, and an *h*-index of 60 is genuinely exceptional [11].

### 2.6 Getting noticed in science and the types of articles one can publish

*“If you know you are on the right track, if you have this inner knowledge, then nobody can turn you off. no matter what they say.” Barbara McClintock*

Over the years, one hears about the best way to be noticed in your scientific field. In the beginning, just attending a research meeting and presenting a poster will be okay. However, as you move up in one’s level of recognition in your chosen field, the hope is that you will be giving a talk (or someone in your laboratory group), chairing a session, or helping to plan the next Conference. The author’s advice from a senior sage scientist was to attend all the important meetings in your field, bring your laboratory group for the experience, and submit your best work to be presented. When submitting an abstract, always choose oral presentation instead of poster presentation; over time, it will make a difference if one is speaking in a room compared to just giving a poster presentation. The author valued this sort of advice. Before the COVID-19 pandemic, it mattered to attend several meetings a year, get noticed, and renew friendships and collaborations as a secondary benefit.

One of the author’s senior advisors once commented that publishing a methods article or a review article would typically get more views than just reporting science. Furthermore, another senior scientist said that book chapters were also good (even if non-peer-reviewed) because these books would/could be seen by many people over a long time. Interestingly, these suggestions were all valuable. Of course, not everyone will write a methods/methodology-type article. However, review articles in one’s field are always in demand. The author’s three most highly cited articles were either a methods article or a review article reported by Google Scholar. In contrast, the fourth most highly cited article was a science/research article, and they are as follows:

**(Cited 1,568 times)** Church et al. “Spectrophotometric assay using o-phthaldialdehyde for determination of proteolysis in milk and isolated milk proteins.” *Journal of Dairy Science* 66, no. 6 (1983): 1219–1227 [12].

**(Cited 1,480 times)** Silverman et al. “The serpins are an expanding superfamily of structurally similar but functionally diverse proteins.” *Journal of Biological Chemistry* 276, 36 (2001): 33293–33296 [13].

**(Cited 390 times)** Rau et al. “Serpins in thrombosis, hemostasis and fibrinolysis.” *Journal of Thrombosis and Haemostasis* 5 (2007): 102–115 [14].

**(Cited 246 times)** Church et al. “Antithrombin activity of fucoidan: the interaction of fucoidan with heparin cofactor II, antithrombin III, and thrombin.” *Journal of Biological Chemistry* 264, no. 6 (1989): 3618–3623 [15].

One will be asked to write review articles, book chapters, and commentaries during an academic career. The perfect places to seek a first author for such work are the graduate students and postdoctoral fellows in one’s laboratory group. Such examples of review articles where the first author was either a graduate student or postdoctoral fellow are given by Pratt and Church [16], Rau et al. [14], Carter and Church [17], Gramling and Church [18], Rau et al. [19], Rein et al. [20], Rein-Smith and Church [21], and Chappell and Church [22]. In contrast, examples of book chapters include Pratt et al. [23], Shirk et al. [24], and Cardenas et al. [25]. In addition, an article dealing with methods includes Bauman et al. [26], whereas commentaries were Beaulieu and Church [27] and Rein et al. [28].

### 2.7 Changing the direction of one’s laboratory research

*“Science means constantly walking a tightrope between blind faith and curiosity; between expertise and creativity; between bias and openness; between experience and epiphany; between ambition and passion; and between arrogance and conviction – in short, between an old today and a new tomorrow.” Henrich Rohrer*

**Figure 1:**
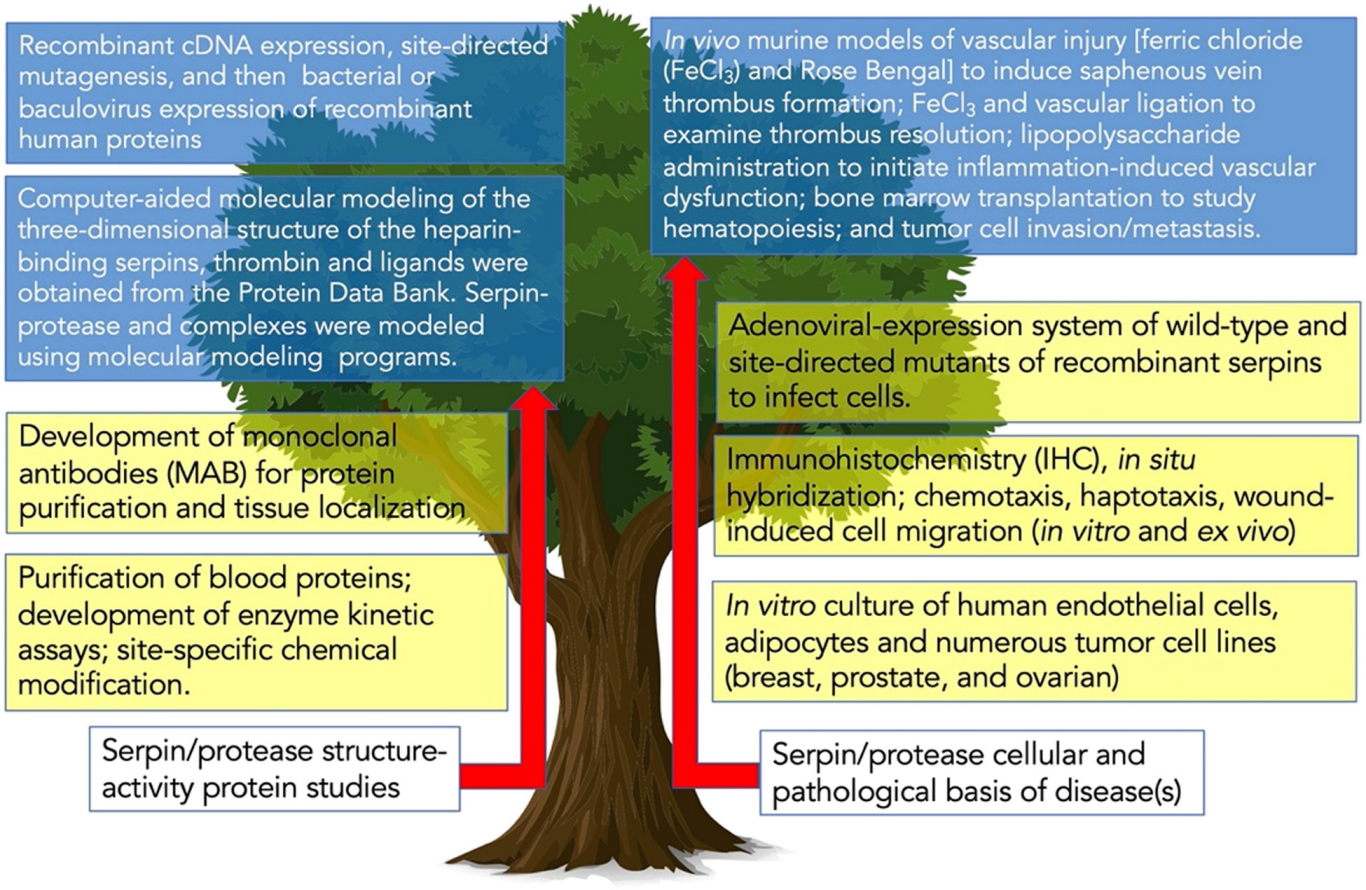
Tree of science from the author’s laboratory. On the bottom are the two significant types of science issues we studied. Moreover, progressing upward in yellow highlights are given the two or three levels of scientific complexity of the methodology we used. Finally, it culminated using the most complex and time-consuming techniques in blue highlight.

The laboratory exists based on one’s science, training, the background of the group, and the overall goals and hypotheses being tested. To stay current, a research laboratory must stay upfront and work with the appropriate tools and techniques. It reminds the author of a tree growing through time in that it is constantly growing upward and branching with each year. Such is the science in one’s laboratory. These decisions are important as next year’s growth ring depends on the tree. Given below are some key branching points on the laboratory’s tree of science (to fully appreciate the author’s work, see the following Google Scholar publication file [29]); the technological advances made in the laboratory are illustrated in Figure 1.

Early in the author’s career, we spent most of our time purifying proteins from human blood [30,31,32]. Instead of focusing our time on the primary heparin-binding serpin named antithrombin, the laboratory compared other lesser well understood serpins (heparin cofactor II and protein C inhibitor) to antithrombin [14,33,34]. To identify essential amino acid residues in serpins and proteases for structure–activity studies, the author’s laboratory moved from site-specific chemical modification studies [35,36,37] to site-directed mutagenesis studies by developing and expressing recombinant proteins [36,38,39,40,41,42,43].

We discovered some novel biological properties for heparin cofactor II, specifically, chemotactic activity [44,45], which prompted us to study the cellular and pathological basis of serpins in disease (*in vitro* and *ex vivo*), specifically vascular biology/wound healing [46,47,48,49,50,51] and cancer biology [52,53,54,55,56,57]. Interestingly, the majority of the cancer biology work was centered on another serpin named plasminogen activator inhibitor-1 (PAI-1) [17,18,58]. The work on PAI-1 was done using both stable transfection and adenoviral gene delivery expressing wild-type and non-inhibitory PAI-1 mutants into various tumor cell lines, and using siRNA to silence the naturally synthesized PAI-1 by tumor cells [53,54,55,56,59,60,61]. The author’s laboratory also studied the proteases being targeted, especially thrombin [62,63], activated protein C [64], and urokinase/tissue plasminogen activator [65]. Furthermore, we were fortunate to be able to study RNA aptamers to (pro)thrombin [66,67,68] and branched into the heparin-binding properties of lactoferrin [69,70]. From these collective studies, we began studying *in vivo* models of vascular injury for thrombosis [71,72] and with collaborators for cancer invasion/metastasis [61,73].

Our approach to research on serpins and proteases was considered innovative. Someone once said we did “structural pathobiology” (combining basic protein chemistry/structure-activity relationships with the more disease/tissue- and cell-based pathology research). We were fortunate to have many successful collaborations and interactions with laboratories across the world [13,20,36,42,48,49,50,51,62,64,67,68,74,75,76,77,78,79,80,81,82,83,84,85].

### 2.8 Intellectual property rights and patents

*“Every brilliant experiment, like every great work of art, starts with an act of imagination.” Jonah Lehrer*

Applying one’s research may create new experiences and work for your laboratory. The crossing-over of one’s idea outside of academia may lead one into the realm of intellectual property rights and patents. If this happens, your university will likely have an Office of Technology Development. They will work with your laboratory to report an invention, cover the costs for a patent attorney to compose and submit the patent paperwork, promote the reported invention, and return any potential profit from the invention (from funds to the laboratory and even linking investors to initiating a startup company). The author never envisioned patents evolving from his research; however, from the six invention reports submitted to the university, two patents were eventually received:

Patent No. 5712247 entitled “Use of Lactoferrin to Modulate and/or Neutralize Heparin Activity” was issued by the US Patent Agency to Hai-feng Wu (postdoctoral fellow), Frank Church, and UNC-CH (1998) [86]. and Patent No. 6207419 entitled “Thrombin Inhibitory Agents” was issued by the US Patent Agency to Susannah Bauman (graduate student), Frank Church. and UNC-CH (2001) [87].

### 2.9 Sharing and supporting science with others matter

*“There is a single light of science, and to brighten it anywhere is to brighten it everywhere.” Isaac Asimov*

Sharing one’s science with others is a powerful endeavor. Yet, interestingly, there is no best formula for a laboratory group. Walk down the hallway of any science building on any campus, and every group will be different from the next one. Likewise, laboratory groups differ in size, types of laboratory group members, and science performed. Notably, the common thread with successful laboratories is the laboratory director supporting their laboratory group members, regardless of their size.

A usual expectation from a laboratory group member is likely a supportive letter of recommendation. The support the laboratory director writes for that group member may be the most important letter of recommendation they need for any program or school or whatever type of position. The author has been told that he had three kinds of letters. The “C-type letter” was a descriptive and supportive letter for a student in a class. The “B-type letter” was a more supportive letter for a student from a class that had become better known to the author. However, the “A-type letter” was reserved for the laboratory group member, and it originated in a special place from the author. This letter was very supportive and much longer with detailed information that helped the student’s quest for a future degree/position. Therefore, if the most demanding task in running a laboratory group is composing a strongly supportive letter of recommendation, it is worth the effort to form a laboratory group.

## 3 Memories of a research laboratory group

### 3.1 Reunited through publishing an article together

*“The good thing about science is that it’s true whether or not you believe in it.” Neil deGrasse Tyson*

Recently, we had a manuscript accepted for publication [4]. Notably, three of my four coauthors were former research laboratory members (Scott Cooper, Yolanda Fortenberry, and Laura Glasscock). In addition, I taught Rebecca Hite as an undergraduate at UNC-CH. Furthermore, it started me reflecting on the value of a laboratory group from a historical, practical, and memorial standpoint. Thus, the reason for writing this commentary.

### 3.2 Your credibility is largely derived from contributions made by the laboratory group

*“Live neither in the past nor in the future, but let each day’s work absorb your entire energies, and satisfy your widest ambition.” Sir William Osler to his students (1849*–*1919)*

The legacy of one’s career and the lasting features of one’s science contributions reside in each person that worked in one’s laboratory. They added to your science story, and you watched them grow during their training. The PI was present while their scientific confidence emerged. One was witnessing the development of their future research philosophy. One may have also spent a considerable amount of time recruiting them to the laboratory, supporting them, or helping them sustain themselves. Likely, one had identified critical projects for them to work on that must get done and other preliminary ideas as building blocks of the future. If these new ideas worked, a whole new direction of science could evolve. And then they are gone (so soon) to pursue a postdoctoral fellowship, a position teaching, or to lead their laboratory.

However, by having been a part of your laboratory, they brought a part of you with them as they left; they had an imprint of you/your laboratory in wherever they were now based. In that way, every person you trained and helped develop as a scientist has a token reflectance of your scientific philosophy embedded in their own laboratory environment. The author brought such invisible imprints to his laboratory from the training and advice in graduate school (MS and PhD) and postdoctoral fellowship.

### 3.3 Laboratory group: Another form of family

*“Science is a way of thinking much more than it is a body of knowledge.” Carl Sagan*

Many may believe that a laboratory group is just a collection of people brought together by the PI to perform science. That is pretty much a typical reason. By contrast, the author always considered the laboratory group as a form of family. Why? Because one spends so much time together, and everyone is on the same side in performing the laboratory’s science. Although one person is the laboratory leader/PI/director, the laboratory personnel are colleagues and more than just employees. At times, their problems become your problems, although there were also many successes along the way. Furthermore, their accomplishments are evident in their attitude and work ethic. Importantly, their willingness to help their laboratory group colleagues further cements their worth to you and the laboratory group.

And thus, one’s laboratory family grows and changes with time, students and postdocts and the like come into one’s laboratory group, pass through, and work together, and then the family changes, and new folks start the process over. The author has now been on the Department of Pathology and Laboratory Medicine faculty at the University of North Carolina at Chapel Hill for 36 years after completing 3 years of postdoct training and 1 year as a non-tenure research assistant professor. As a basic biomedical researcher, the author has had a wonderful and enriching academic research career that helped train over 100 scientists [17 graduate students, 11 postdoctoral fellows, 64 undergraduates, and 17 medical students (not including all the numerous research technicians and visiting scholars)].

### 3.4 Ten words that will help one’s career

*“An experiment is a question which science poses to Nature and a measurement is the recording of Nature’s answer.” Max Planck*

At the beginning of the author’s academic career, he wrote on a piece of paper ten words that all began with the letter “P.” These words were focused on the mindset of how to achieve/sustain success in the world of medical academics/research in a university setting. What is the worth of these P-words? They can focus your mind and effort while you advance/survive/navigate/succeed through your career. At various times during your career, some words may take precedence depending on the situation. However, consider the words like a song’s melody; they will all significantly contribute to the symphony of one’s work-life. There is no doubt that there are many other words one could cite that help one navigate work, which allow one to succeed in one’s career. However, may this list of ten P-words help you focus and achieve further in your professional career (they are listed alphabetically followed by definition from https://www.thefreedictionary.com/).

1. Passionate (capable of, having, or dominated by powerful emotions)
2. Patient (tolerant; understanding)
3. Perseverance (continued steady belief or efforts, withstanding discouragement)
4. Persistent (continuance of an effect after the cause is removed)
5. Positivity (characterized by or displaying certainty, acceptance, or affirmation)
6. Power (the ability or capacity to act or do something effectively)
7. Prepared (to make ready beforehand for a specific purpose)
8. Principled(s) (based on, marked by, or manifesting principle)
9. Productive (effective in achieving specified results)
10. Purposeful (determined; resolute)

## 4 Conclusion

*“Life is not easy for any of us. But what of that? We must have perseverance and above all confidence in ourselves. We must believe that we are gifted for something, and that this thing, at whatever cost, must be attained.” Marie Curie*

Laboratory groups are all different, and they revolve around the thinking and ideas of the laboratory members at that time. Therefore, the author has great memories of everyone at every stage of their career. The exciting event was when former laboratory group members from different times met (usually at various national/international meetings); they shared a common bond and interest; it is always great to witness.

**Figure 2:**
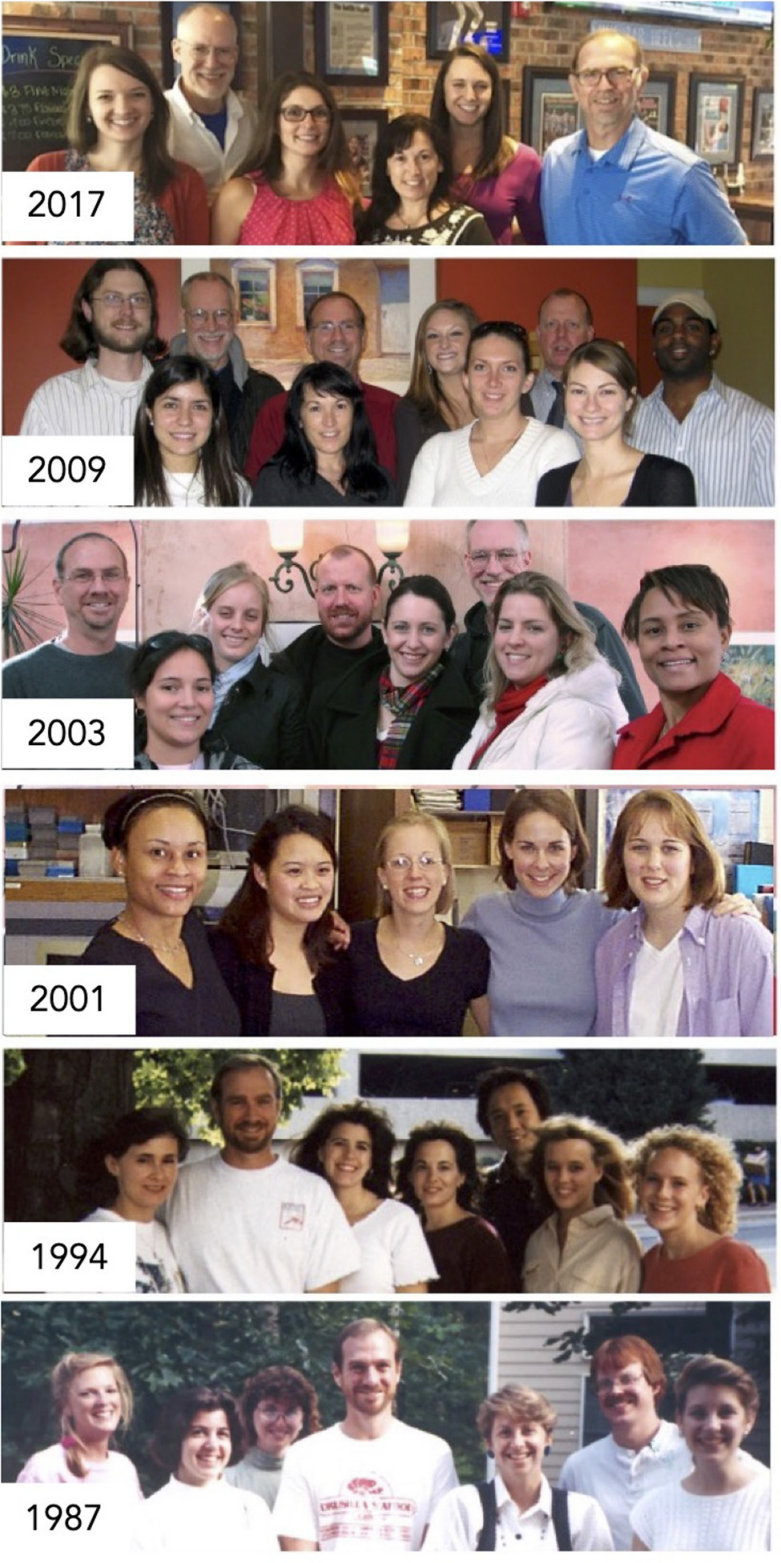
Laboratory Group Pictures (representative pictures from 1987–2017; we closed the lab in 2019). It is probably impossible to have a photo of every person that has ever worked in your research laboratory. However, for over 30 years, we functioned as a laboratory group. Although there are no “owner’s manuals” for running, recruiting, and operating a laboratory group, we certainly learned a lot along the way.

The laboratory space will be made up of walls, equipment, benches, and desks; this is the foundation. However, this only represents science-waiting-to-happen. When the laboratory group occupies this space, experiments are being performed to test a hypothesis, with voices talking in support of one another; that is when one knows this is a functional laboratory group. Ultimately, the laboratory group helped define the author’s academic career over the years. It indeed guided him through the research process. In closing, the author is most thankful for all the past members of the laboratory (Figure 2).
